# Isogenic human trophectoderm cells demonstrate the role of *NDUFA4* and associated variants in ZIKV infection

**DOI:** 10.1016/j.isci.2023.107001

**Published:** 2023-05-29

**Authors:** Liuliu Yang, Yuling Han, Ting Zhou, Lauretta A. Lacko, Mohsan Saeed, Christina Tan, Ron Danziger, Jiajun Zhu, Zeping Zhao, Clare Cahir, Alice Maria Giani, Yang Li, Xue Dong, Dorota Moroziewicz, The NYSCF Global Stem Cell Array® Team, Daniel Paull, Zhengming Chen, Aaron Zhong, Scott A. Noggle, Charles M. Rice, Qibin Qi, Todd Evans, Shuibing Chen

**Affiliations:** 1 Department of Surgery, Weill Cornell Medicine, 1300 York Avenue, New York, NY 10065, USA; 2 Center for Genomic Health, Weill Cornell Medicine, 1300 York Avenue, New York, NY 10065, USA; 3 Stem Cell Research Facility, Memorial Sloan Kettering Cancer Center, 1275 York Avenue, New York, NY 10065, USA; 4 Laboratory of Virology and Infectious Disease, The Rockefeller University, New York, NY 10065, USA; 5 Department of Biochemistry & Cell Biology, Boston University Chobanian and Avedisian School of Medicine, Boston, MA 02118, USA; 6 National Emerging Infectious Diseases Laboratories (NEIDL), Boston University, Boston, MA 02118, USA; 7 Department of Epidemiology & Population Health, Albert Einstein College of Medicine, 1300 Morris Park Avenue, Bronx, NY 10461, USA; 8 The New York Stem Cell Foundation Research Institute, 619 West 54th Street, 3Road Floor, New York, NY 10019, USA; 9 Department of Population Health Sciences, Weill Cornell Medicine, 1300 York Avenue, New York, NY 10065, USA

**Keywords:** Stem cells research, Virology

## Abstract

Population-based genome-wide association studies (GWAS) normally require a large sample size, which can be labor intensive and costly. Recently, we reported a human induced pluripotent stem cell (hiPSC) array-based GWAS method, identifying NDUFA4 as a host factor for Zika virus (ZIKV) infection. In this study, we extended our analysis to trophectoderm cells, which constitute one of the major routes of mother-to-fetus transmission of ZIKV during pregnancy. We differentiated hiPSCs from various donors into trophectoderm cells. We then infected cells carrying loss of function mutations in *NDUFA4*, harboring risk versus non-risk alleles of SNPs (rs917172 and rs12386620) or having deletions in the *NDUFA4 cis*-regulatory region with ZIKV. We found that loss/reduction of NDUFA4 suppressed ZIKV infection in trophectoderm cells. This study validated our published hiPSC array-based system as a useful platform for GWAS and confirmed the role of NDUFA4 as a susceptibility locus for ZIKV in disease-relevant trophectoderm cells.

## Highlights

• Trophectoderm from various hiPSCs show variable susceptibility to ZIKV
• A deficiency of NDUFA4 in trophectoderm causes reduced ZIKV infection
• Trophectoderm carrying risk alleles show increased ZIKV infection
• Deletion of NDUFA4 regulatory elements leads to decreased ZIKV infection

## Graphical abstract

**Figure:**
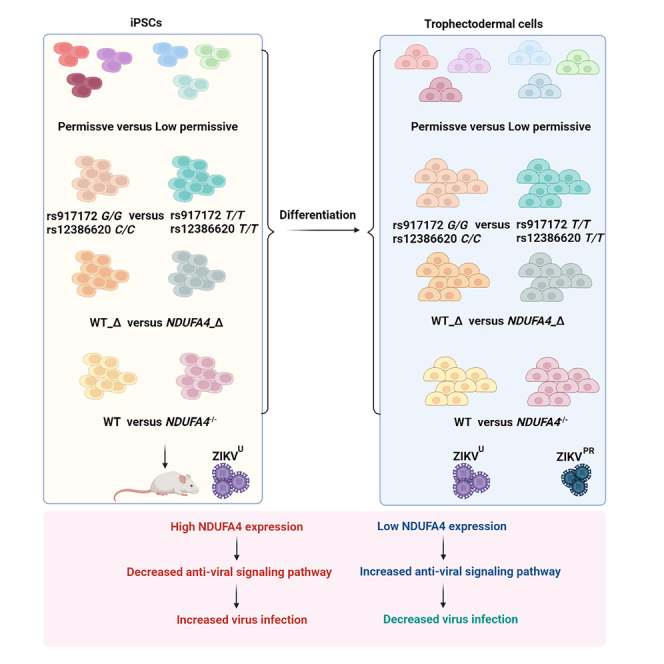

## Introduction

Genome-wide association studies (GWAS) have been successful at identifying specific genetic variations that associate with particular diseases.^1^ GWAS are typically based on human cohorts, members of whom have diverse lifestyles, variable co-morbidities, and different environmental exposures. Human population based GWAS, therefore, usually requires recruitment of a large cohort, which is expensive and labor-intensive.^2^ Recently, we performed GWAS using a human induced pluripotent stem cell (hiPSC) array, containing 77 hiPSC lines with distinct genetic backgrounds. We identified NDUFA4 as an important host dependency factor for Zika virus (ZIKV) replication in hiPSCs and hiPSC-derived cerebral organoids.^3^ In this study, we explore the relevance of NDUFA4 to ZIKV infection in trophectoderm cells, which constitute a route of ZIKV transmission from mother to fetus and therefore represent an important disease-relevant cell type.

ZIKV is a member of the Flaviviridae family. Vertical transmission of ZIKV from mother to fetus is linked to increased incidence of congenital ZIKV syndrome in babies, including microcephaly, congenital malformation, and fetal demise.^4^^,^^5^^,^^6^^,^^7^ Among mothers with confirmed or suspected infection of ZIKV, about 5% give birth to babies suffering from ZIKV-associated birth defects. Unfortunately, there are no vaccines or drugs to prevent or treat ZIKV infection. Therefore, it is urgent to explore the mechanism and find a potential treatment for ZIKV infection during pregnancy.^8^ The outer layer of the blastocyst-stage embryo is trophectoderm, which is the precursor of all trophoblast cells of the placenta. Recent studies using pre-implantation human and mouse embryos demonstrated that trophectoderm cells can be infected with and propagate ZIKV, causing neural progenitor cell death.^9^ In addition, ZIKV can infect the trophectoderm of *in vitro* fertilized rhesus macaque embryos, which negatively impacts the attachment, growth, and survival of the blastocysts.^10^ Studies also showed that ZIKV can infect both cytotrophoblast and syncytiotrophoblast derived from placental villi at term and colonies of trophoblast differentiated from embryonic stem cells (ESC).^11^ ZIKV infection of the trophoblast cells affects cell migration, metabolism and immune homeostasis control,^12^ endoplasmic reticulum stress, apoptosis^13^ and pyroptosis.^14^ Type III interferons produced by human placental trophoblasts protect the non-placental cells against ZIKV infection.^15^ In addition, ZIKV infected trophoblasts become a target of decidual natural killer cells, which release their granules and kill ZIKV-infected trophoblasts.^16^ ZIKV infected decidual cells exaggerate the infection of trophoblasts.^17^ Here, we systematically examine the impact of NDUFA4 and associated SNPs on the permissiveness of trophectoderm cells to ZIKV infection. In addition, we created a xenograft model to examine the impact of the loss of NDUFA4 on ZIKV infection *in vivo*. Together, our studies show that NDUFA4 and associated SNPs affect the ZIKV infection of trophectoderm cells *in vitro* and *in vivo*.

## Results

### Trophectoderm cells originating from different hiPSC lines show variable permissiveness to ZIKV infection

**Figure 1:**
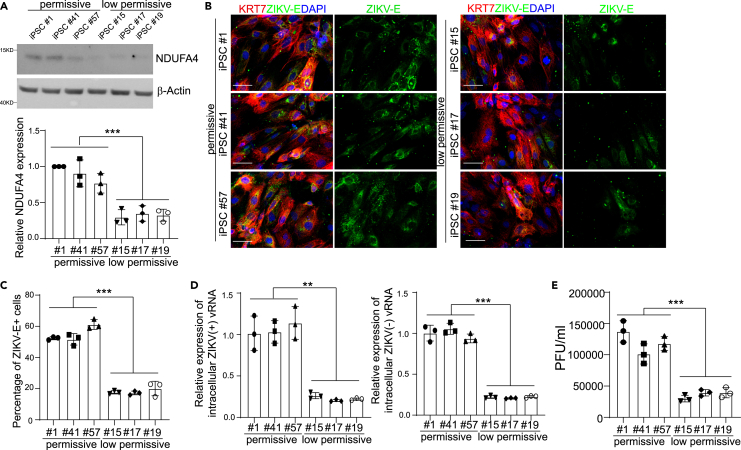
Trophectoderm cells derived from different hiPSC lines display distinct ZIKV permissiveness (A) Western blotting analysis and the quantification of NDUFA4 protein expression in trophectoderm cells derived from permissive cell lines: iPSC #1, iPSC #41 and iPSC #57 and low permissive cell lines: iPSC #15, iPSC #17 and iPSC #19. β-Actin was used as a loading control. (B and C) Representative confocal images (B) and the quantification (C) of ZIKV-E staining in KRT7^+^ trophectoderm cells derived from permissive cell lines: iPSC #1, iPSC #41 and iPSC #57 and low permissive cell lines: iPSC #15, iPSC #17 and iPSC #19 at 72 hpi (ZIKV^PR^, MOI = 1). Scale bar = 50 μm. (D) qRT-PCR analysis of (+) or (−) ZIKV vRNA strands in trophectoderm cells derived from permissive cell lines: iPSC #1, iPSC #41 and iPSC #57 and low permissive cell lines: iPSC #15, iPSC #17 and iPSC #19 at 72 hpi (ZIKV^PR^, MOI = 1). (E) Viral titers of ZIKV in the supernatant of trophectoderm cells derived from permissive cell lines: iPSC #1, iPSC #41 and iPSC #57 and low permissive cell lines: iPSC #15, iPSC #17 and iPSC #19 (ZIKV^PR^, MOI = 1) quantified by plaque assay. Data are representative of at least three independent experiments. For p values, we averaged the 3 technical replicates within each cell line, then used the averages for an unpaired two-tailed Student’s *t* test. ∗∗p < 0.01, and ∗∗∗p < 0.001. See also Figure S1.

In our previous study, we screened 77 hiPSC lines for their ability to support ZIKV infection, identifying 39 “low-permissive” lines and 38 “permissive” lines.^3^^,^^18^To determine whether trophectoderm cells derived from these hiPSC lines also show differential permissiveness to ZIKV infection, three permissive lines (hiPSC lines #1, #41, and #57) and three low-permissive lines (hiPSC lines #15, #17 and #19) were differentiated into trophectoderm cells^3^^,^^9^ (Figure S1A). All hiPSC lines showed a similar capacity to generate keratin-7-positive (KRT7^+^) trophectoderm cells (∼90%, Figures S1B and S1C). We performed several assays to test the permissiveness of trophectoderm cells to ZIKV infection. Consistent with the expression of NDUFA4 in hiPSCs, trophectoderm cells derived from permissive hiPSC lines expressed higher levels of NDUFA4 compared to those derived from low-permissive lines (Figure 1A). As shown in Figures 1B and 1C, the percentage of ZIKV-E^+^ cells was significantly higher for trophectoderm cells derived from permissive hiPSC lines compared to those derived from low-permissive lines (ZIKV^PR^, Puerto Rico strain, PRVABC59). In addition, significantly higher levels of both (+) and (−) strands of ZIKV vRNA were detected in trophectoderm cells derived from permissive hiPSC lines (Figure 1D). A similar pattern was seen when the infection efficiency was monitored by measuring the release of infectious particles in the supernatant of infected cells. (Figure 1E). In all, these findings indicate that ZIKV infection in trophectoderm cells follows the same pattern seen in their progenitor hiPSCs.

**Figure 2:**
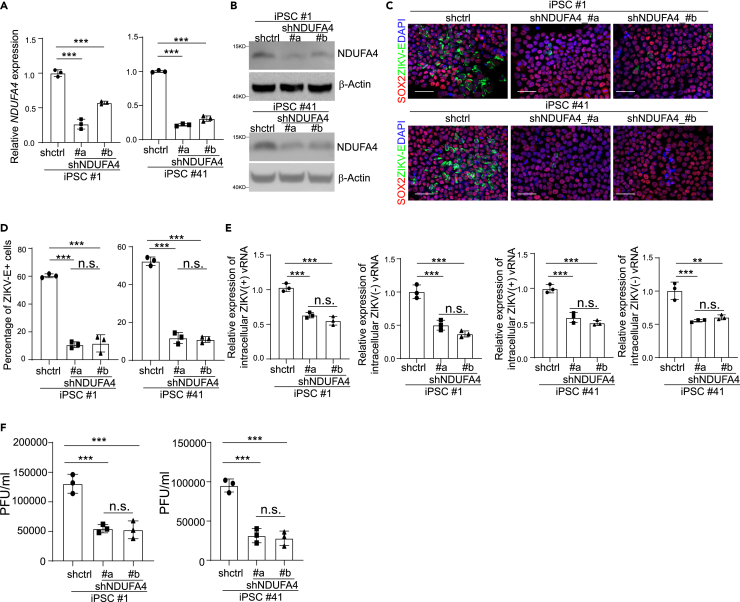
Knockdown of NUDFA4 decreases ZIKV infection permissiveness (A) qRT-PCR analysis of *NDUFA4* mRNA expression levels in the NDUFA4 knockdown cell lines of iPSC #1 (shctrl, shNDUFA4 #a, shNDUFA4 #b) and iPSC 41 (shctrl, shNDUFA4 #a, shNDUFA4 #b). The shctrl is a scrambled shRNA. (B) Western blotting analysis of NDUFA4 protein expression levels in the NDUFA4 knockdown cell lines of iPSC #1 (shctrl, shNDUFA4 #a, shNDUFA4 #b) and iPSC #41 (shctrl, shNDUFA4 #a, shNDUFA4 #b). β-Actin was used as a loading control. (C and D) Representative confocal images (C) and the quantification (D) of SOX2 and ZIKV-E staining in NDUFA4 knockdown cell lines of iPSC #1 (shctrl, shNDUFA4 #a, shNDUFA4 #b) or iPSC #41 (shctrl, shNDUFA4 #a, shNDUFA4 #b) at 72 hpi (ZIKV^PR^, MOI = 1). Scale bar = 50 μm. (E) qRT-PCR analysis of (+) and (−) ZIKV vRNA strands in NDUFA4 knockdown cell lines of iPSC #1 (shctrl, shNDUFA4 #a, shNDUFA4 #b) or iPSC #41 (shctrl, shNDUFA4 #a, shNDUFA4 #b) at 72 hpi (ZIKV^PR^, MOI = 1). (F) Viral titers of ZIKV in the supernatant of NDUFA4 knockdown cell lines of iPSC #1 (shctrl, shNDUFA4 #a, shNDUFA4 #b) or iPSC #41 (shctrl, shNDUFA4 #a, shNDUFA4 #b) at 72 hpi (ZIKV^PR^, MOI = 1) quantified by plaque assay. Data are representative of at least three independent experiments. p values were calculated by one-way ANOVA followed by a Dunnett’s post hoc test with a common control for multiple testing correction. n.s. no significance and ∗∗∗p < 0.001. See also Figure S2.

Our previous study established a link between NDUFA4 expression and permissiveness to ZIKV infection using one iPSC line.^3^ To further validate these findings with multiple iPSC lines from different gender and race backgrounds, we knocked down NDUFA4 in two permissive iPSC lines (#1 and #41) using shRNAs. qRT-PCR (Figure 2A) and western blotting assays (Figure 2B) confirmed the reduction of NDUFA4 levels in iPSCs expressing shNDUFA4 #a or shNDUFA4 #b. Following ZIKV infection, the iPSCs carrying shNDUFA4 #a or shNDUFA4 #b showed a lower percentage of ZIKV-E^+^ cells at 72 hpi (ZIKV^PR^: Figures 2C and 2D; ZIKV^U^: West Africa strain, MR766: Figures S2A and S2B) and decreased levels of ZIKV (+) and (−) vRNA strands (ZIKV^PR^: Figure 2E; ZIKV^U^: Figure S2C) compared to control iPSCs carrying scrambled shRNA. Consistently, the decreased yields of infectious ZIKV were detected in the supernatant of the iPSCs carrying shNDUFA4 #a or shNDUFA4 #b (ZIKV^PR^: Figure 2F; ZIKV^U^: Figure S2D).

### Deficiency of NDUFA4 in trophectoderm cells leads to decreased ZIKV infection

**Figure 3:**
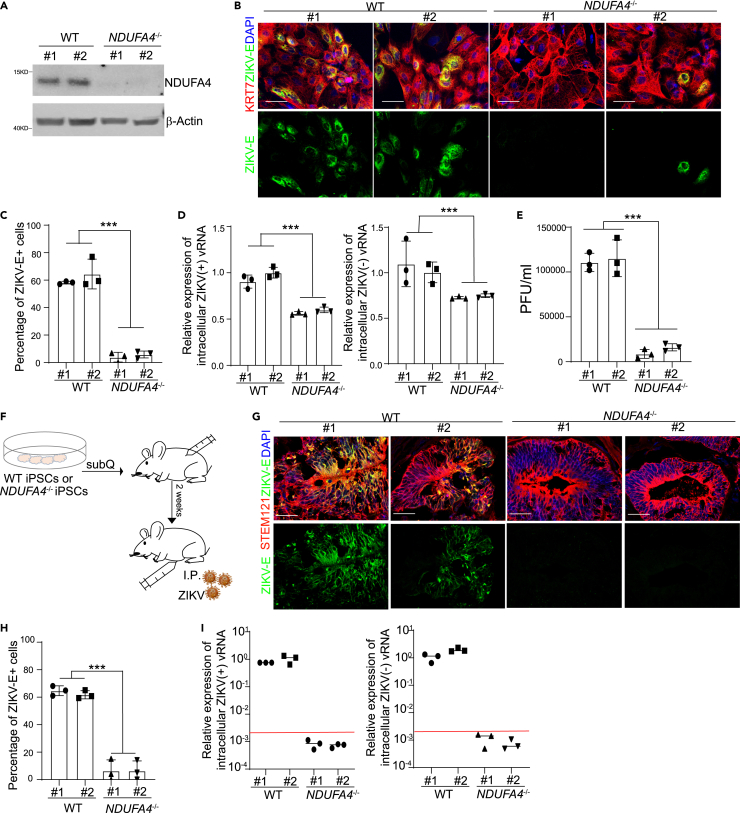
Deficiency of NDUFA4 leads to decreased ZIKV infection *in vitro* and *in vivo* (A) Western blotting analysis of NDUFA4 protein expression in trophectoderm cells derived from WT or *NDUFA4*^*−/−*^ hiPSCs. β-Actin was used as a loading control. (B and C) Representative confocal images (B) and the quantification (C) of ZIKV-E staining in KRT7^+^ trophectoderm cells derived from WT or *NDUFA4*^*−/−*^ hiPSCs at 72 hpi (ZIKV^PR^, MOI = 1). Scale bar = 50 μm. (D) qRT-PCR analysis of (+) or (−) ZIKV vRNA strands in trophectoderm cells derived from WT or *NDUFA4*^*−/−*^ hiPSCs at 72 hpi (ZIKV^PR^, MOI = 1). (E) Viral titers of ZIKV in the supernatant of trophectoderm cells derived from WT or *NDUFA4*^*−/−*^ hiPSCs at 72 hpi (ZIKV^PR^, MOI = 1) quantified by plaque assay. (F) Scheme of the *in vivo* xeno-transplantation and ZIKV infection. (G and H) Representative images (G) and the quantification (H) of ZIKV-E and STEM121 staining in WT or *NDUFA4*^*−/−*^ hiPSCs derived xenografts at 5 dpi (ZIKV^U^, 5 × 10^5^ PFU). Scale bar = 50 μm. (I) qRT-PCR analysis of (+) or (−) ZIKV vRNA strands in WT or *NDUFA4*^*−/−*^ hiPSCs derived xenografts at 5 dpi (ZIKV^U^, 5 × 10^5^ PFU). The viral RNA level in *NDUFA4*^*−/−*^ hiPSCs derived xenografts is below detection. The red line indicates the detection limit. Data are representative of at least three independent experiments. p values were calculated by two-way ANOVA analysis; ∗∗∗p < 0.001. See also Figure S3.

In our previous study, we generated *NDUFA4*^*−/−*^ iPSC lines using a CRISPR-based gene knockout strategy.^3^ To determine the role of NDUFA4 in ZIKV infection in trophectoderm cells, WT and *NDUFA4*^*−/−*^ iPSCs were first differentiated into trophectoderm cells. Western blot analysis confirmed the loss of NDUFA4 expression in *NDUFA4*^*−/−*^ trophectoderm cells (Figure 3A). WT and *NDUFA4*^*−/−*^ iPSCs demonstrated similar capacities to differentiate into KRT7^+^ trophectoderm cells (Figures S3A and S3B), suggesting that the loss of NDUFA4 does not affect iPSC differentiation to trophectoderm cells. Following infection of WT or *NDUFA4*^*−/−*^ iPSC-derived trophectoderm cells with ZIKV, we found a lower percentage of ZIKV-E^+^ cells in *NDUFA4*^*−/−*^ trophectoderm cells (ZIKV^PR^: Figures 3B and 3C; ZIKV^U^: Figures S3C and S3D). Consistent with this observation, we found lower levels of (+) and (−) strand ZIKV vRNA in *NDUFA4*^*−/−*^ trophectoderm cells (ZIKV^PR^: Figure 3D; ZIKV^U^: Figure S3E), as well as lower viral titers in the supernatant of *NDUFA4*^*−/−*^ trophectoderm cells (ZIKV^PR^: Figure 3E; ZIKV^U^: Figure S3F).

To examine the permissiveness of *NDUFA4*^*−/−*^ and WT hiPSCs to ZIKV infection *in vivo*, hiPSCs were transplanted subcutaneously into immune-deficient SCID-beige mice, and the mice were inoculated with ZIKV^U^ by intraperitoneal injection two weeks post-transplantation (Figure 3F). Five days post infection, the xenografts were collected and stained with antibodies against human antigen (STEM121) and ZIKV-E. The percentage of ZIKV-E^+^ cells in *NDUFA4*^*−/−*^ iPSC-derived xenografts was significantly lower compared with WT hiPSC-derived xenografts (Figures 3G and 3H). We also found decreased viral RNA in *NDUFA4*^*−/−*^ iPSC-derived xenografts examined by qRT-PCR (Figure 3I).

### Trophectoderm cells derived from hiPSC lines carrying the risk alleles of rs917172 and rs12386620 show increased ZIKV infection

**Figure 4:**
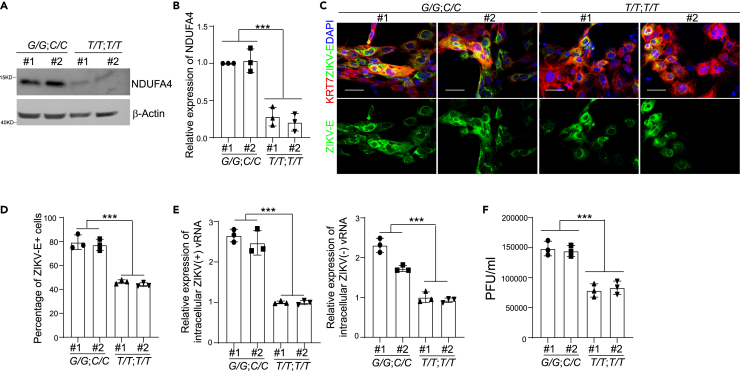
Trophectoderm cells derived from iPSCs with risk alleles of rs917172 and rs12386620 show increased sensitivity to ZIKV infection (A and B) Western blotting analysis (A) and quantification (B) of NDUFA4 protein expression in trophectoderm cells derived from hiPSCs carrying risk (*G/G*; *C/C*) or non-risk (*T/T*; *T/T*) alleles. β-Actin was used as a loading control. (C and D) Representative confocal images (C) and the quantification (D) of ZIKV-E staining in KRT7^+^ trophectoderm cells derived from hiPSCs carrying risk (*G/G*; *C/C*) or non-risk (*T/T*; *T/T*) alleles at 72 hpi (ZIKV^PR^, MOI = 1). Scale bar = 50 μm. (E) qRT-PCR analysis of (+) or (−) ZIKV vRNA strands in trophectoderm cells derived from hiPSCs carrying risk (*G/G*; *C/C*) or non-risk (*T/T*; *T/T*) alleles at 72 hpi (ZIKV^PR^, MOI = 1). (F) Viral titers of ZIKV in the supernatant of trophectoderm cells derived from hiPSCs carrying risk (*G/G*; *C/C*) or non-risk (*T/T*; *T/T*) alleles at 72 hpi (ZIKV^PR^, MOI = 1) quantified by plaque assay. Data are representative of at least three independent experiments. p values were calculated by two-way ANOVA analysis; ∗∗∗p < 0.001. See also Figure S4.

Our hiPSC-based GWAS analysis identified several SNPs that are associated with permissiveness to ZIKV infection, including rs917172 and rs12386620 located in an approximately 1 kb region downstream of NDUFA4. Using CRISPR based gene editing, we created isogenic hiPSC lines with risk *G* allele for rs917172 and risk *C* allele for rs12386620. Two hiPSC clones with non-risk *T* alleles for both rs917172 and rs12386620 loci were used as controls. To determine the role of genetic variants in disease-relevant trophectoderm cells, the isogenic hiPSC lines carrying risk alleles (*G/G*; *C/C*) or non-risk alleles (*T/T*; *T/T*) were differentiated into trophectoderm cells. Trophectoderm cells derived from hiPSC lines carrying risk alleles (*G/G*; *C/C*) showed higher NDUFA4 expression than non-risk alleles (*T/T*; *T/T*) (Figures 4A and 4B). The iPSC lines carrying risk alleles (*G/G*; *C/C*) or non-risk alleles (*T/T*; *T/T*) equally efficiently differentiated into KRT7^+^ trophectoderm cells (Figures S4A and S4B). 72h after ZIKV infection, a higher percentage of ZIKV-E^+^ cells were found in trophectoderm cells carrying risk alleles (*G/G*; *C/C*) compared to those carrying non-risk alleles (*T/T*; *T/T*) (ZIKV^PR^: Figures 4C and 4D; ZIKV^U^: Figures S4C and S4D). Similarly, increased levels of ZIKV (+) and (−) vRNA strands (ZIKV^PR^: Figure 4E; ZIKV^U^: Figure S4E) and infectious virus particles (ZIKV^PR^: Figure 4F; ZIKV^U^: Figure S4F) were detected in trophectoderm cells harboring risk alleles (*G/G*; *C/C*) than non-risk alleles (*T/T*; *T/T*).

### Trophectoderm cells carrying deleted *cis*-regulatory elements of NDUFA4 show decreased ZIKV infection

**Figure 5:**
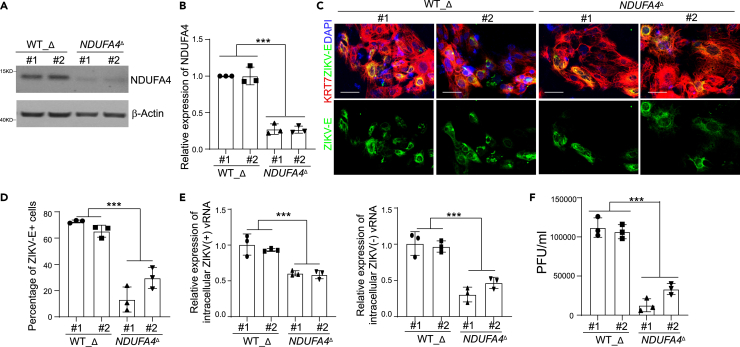
Trophectoderm cells derived from iPSCs with deleted *cis*-regulatory region of *NDUFA4* show decreased ZIKV infection (A and B) Western blotting analysis (A) and quantification (B) of NDUFA4 protein expression in trophectoderm cells derived from WT_Δ or *NDUFA4*^Δ^ hiPSCs. β-Actin was used as a loading control. (C and D) Representative confocal images (C) and the quantification (D) of ZIKV-E staining in KRT7^+^ trophectoderm cells derived from WT_Δ or *NDUFA4*^Δ^ hiPSCs at 72 hpi (ZIKV^PR^, MOI = 1). Scale bar = 50 μm. (E) qRT-PCR analysis of (+) or (−) ZIKV vRNA strands of trophectoderm cells derived from WT_Δ or *NDUFA4*^Δ^ hiPSCs at 72 hpi (ZIKV^PR^, MOI = 1). (F) Viral titers of ZIKV in the supernatant of trophectoderm cells derived from WT_Δ or *NDUFA4*^Δ^ hiPSCs at 72 hpi (ZIKV^PR^, MOI = 1) quantified by plaque assay. Data are representative of at least three independent experiments. p values were calculated by two-way ANOVA analysis; ∗∗∗p < 0.001. See also Figure S5.

Rs917172 and rs12386620 are located in an approximately 1 kb region downstream of NDUFA4. We previously knocked out this putative *cis*-regulatory region in hiPSCs to obtain *NDUFA4*^*Δ*^ cells. To determine whether this regulatory region is associated with permissiveness to ZIKV infection in trophectoderm cells, we differentiated *NDUFA4*^*Δ*^ and WT_Δ hiPSC lines into trophectoderm cells. Trophectoderm cells derived from WT_Δ hiPSC lines showed higher NDUFA4 expression than *NDUFA4*^*Δ*^ hiPSC lines (Figures 5A and 5B). No difference in differentiation potential was seen between *NDUFA4*^*Δ*^ and WT_Δ iPSCs (Figures S5A and S5B). After ZIKV infection, a lower percentage of ZIKV-E^+^ cells were detected in *NDUFA4*^*Δ*^ hiPSC-derived trophectoderm cells compared with WT_Δ hiPSC-derived trophectoderm cells (ZIKV^PR^: Figures 5C and 5D; ZIKV^U^: Figures S5C and S5D). In addition, the levels of ZIKV (+) and (−) vRNA strands were significantly lower in *NDUFA4*^*Δ*^ hiPSC-derived trophectoderm cells compared with WT_Δ hiPSC-derived trophectoderm cells (ZIKV^PR^: Figure 5E; ZIKV^U^: Figure S5E). Consistently, a lower yield of infectious ZIKV was detected in the supernatant of trophectoderm cells derived from *NDUFA4*^*Δ*^ hiPSCs (ZIKV^PR^: Figure 5F; ZIKV^U^: Figure S5F). In summary, these data suggest that the genomic region containing the cluster of SNPs functions as a *cis*-regulatory region for NDUFA4 expression relevant to ZIKV infection of trophectoderm cells.

**Figure 6:**
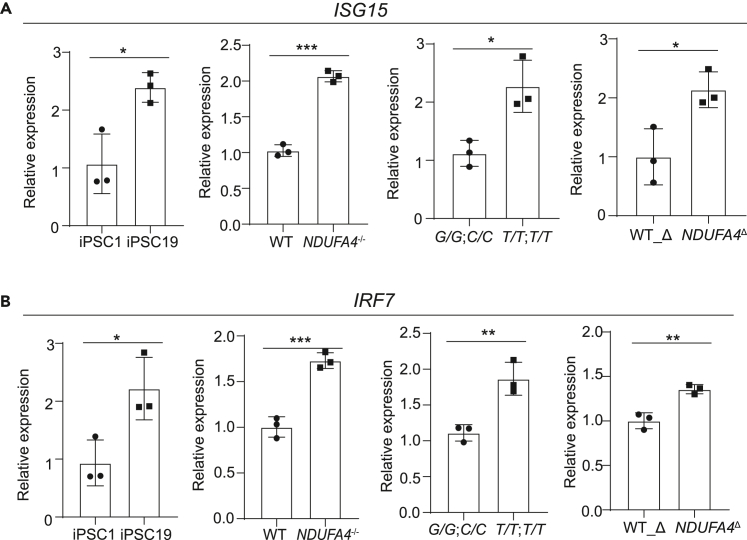
Loss or reduction of NDUFA4 leads to interferon-stimulated gene expression (A and B) qRT-PCR analysis of *ISG15* (A) and *IRF7* (B) mRNA expression levels in trophectoderm cells derived from iPSC #1 v.s. iPSC #19; WT v.s. *NDUFA4*^*−/−*^ hiPSCs; risk (*G/G*; *C/C*) v.s. non-risk (*T/T*; *T/T*) hiPSCs and WT_Δ v.s. *NDUFA4*^Δ^ hiPSCs at 72 hpi (ZIKV^PR^, MOI = 1). Data are representative of at least three independent experiments. p values were calculated by unpaired two-tailed Student’s *t* test; ∗p < 0.05, ∗∗p < 0.01 and ∗∗∗p < 0.001.

Our previous study showed that NDUFA4 deficiency leads to mitochondrial DNA leakage, which can trigger type I interferon signaling in hiPSCs, leading to upregulation of interferon-stimulated genes (ISGs).^3^ To test if NDUFA4 exerts a similar effect in trophectoderm cells, we examined the expression of two ISGs, *ISG15* and interferon regulatory factor 7 (*IRF7*), in hiPSC-derived trophectoderm cells. Increased expression of these ISGs was seen in cells derived from *NDUFA4*^*−/−*^ iPSCs versus WT iPSCs, iPSC lines carrying non-risk (*T/T*; *T/T*) versus risk (*G/G*; *C/C*) alleles, *NDUFA4*^*Δ*^ iPSCs versus WT_Δ iPSCs and iPSC #19 versus iPSC #1 lines under ZIKV infected conditions (Figures 6A and 6B). Altogether, we showed that NDUFA4 deficiency leads to increased type I interferon signaling to limit ZIKV infection in trophectoderm cells.

## Discussion

In our previous study, we conducted a hiPSC-based GWAS and identified NDUFA4 as a locus associated with ZIKV susceptibility.^3^ Through CRISPR-Cas9 technology, we also showed that the SNPs (rs917172 and rs12386620) and a *cis*-regulatory region associated with NDUFA4 expression impact ZIKV infection. Yet, our previous study only focused on the role of NDUFA4 in hiPSCs and cerebral organoids. An advantage of hiPSC-based approach is that these cells can be differentiated into multiple disease-relevant cell types. In this study, we extended our analysis to trophectoderm cells, which act as one of the major routes of vertical transmission of ZIKV during pregnancy. We differentiated WT hiPSC and CRISPR-based gene-edited hiPSC into disease-relevant trophectoderm cells and validated the role of NDUFA4 and its associated variants in ZIKV infection. It is interesting that we detected ZIKV envelope protein in some cells not expressing KRT7 following the knockdown of NDUFA4 or with deletion of the *cis*-regulatory region. We hypothesize that the few non-KRT7-positive cells could be undifferentiated hiPSCs, which were previously reported to be susceptible to ZIKV infection.^3^

NDUFA4 is a mitochondrial complex associated protein. Recent studies reported that NDUFA4 might be a component of the cytochrome *c* oxidase (Balsa et al., 2012; Kadenbach, 2017). Here we found that the loss of NDUFA4 leads to decreased susceptibility to ZIKV infection in trophectoderm cells. Mechanistically, lower expression of NDUFA4 induces mitochondrial stress and leakage of mitochondrial DNA, which can induce upregulation of type-1 interferon signaling, blocking viral infection.^3^^,^^19^ Our data suggest that the trophectoderm cells with relatively high expression levels of NDUFA4 may have lower type I interferon signaling, resulting in the increased capacity for viral replication.

In summary, our study establishes the role of NDUFA4 in ZIKV infection of human trophectoderm cells. We showed that the loss of the NDUFA4 *cis*-regulatory region and the correction of risk-associated SNPs in the NDUFA4 gene led to decreased ZIKV infection in trophectoderm cells. These findings are consistent with our previous report in which we investigated the role of NDUFA4 in hiPSC-derived neuronal cells, suggesting that the function of NDUFA4 is conserved across cell types. Overall, this study highlights the value of our hiPSC-GWAS platform for identifying human genes essential for virus replication in different cellular backgrounds.

### Limitations of the study

In this study, we showed that NDUFA4 and its associated genetic variants have crucial roles in ZIKV infection in trophectoderm cells. Further investigation is required to ascertain if these genetic variants correlate with human susceptibility to infection.

## STAR★Methods

### Key resources table

**Table:** 

REAGENT or RESOURCE	SOURCE	IDENTIFIER
**Antibodies**		

Anti-Flavivirus group antigen [D1-4G2-4-15 (4G2)]	GeneTex	Cat #GTX57154; RRID: AB_2887950
Anti-Flavivirus group antigen [D1-4G2-4-15 (4G2)]	Millipore	Cat #MAB10216-I; RRID: AB_827205
Sox2 Antibody	Cell Signaling	Cat #3579; RRID: AB_2195767
Anti-NDUFA4 antibody	Abcam	Cat #ab129752; RRID: AB_11155881
Anti-Keratin 7 (D1E4) XP®	Cell Signaling	Cat #4465; RRID: AB_11178382
Anti-STEM121 human antigen	Takara	Cat #Y40410; RRID: AB_2801314
Donkey anti-Mouse IgG (H + L) Highly Cross-Adsorbed Secondary Antibody, Alexa Fluor 488	Thermo Fisher Scientific	Cat #A-21202; RRID: AB_141607
Donkey anti-Mouse IgG (H + L) Highly Cross-Adsorbed Secondary Antibody, Alexa Fluor 594	Thermo Fisher Scientific	Cat #A-21203; RRID: AB_141633
Donkey anti-Rabbit IgG (H + L) Secondary Antibody, Alexa Fluor 594 conjugate	Thermo Fisher Scientific	Cat #A-21207; RRID: AB_141637
Donkey anti-Rabbit IgG (H + L) Secondary Antibody, Alexa Fluor 647 conjugate	Thermo Fisher Scientific	Cat #A-31573; RRID: AB_2536183
Donkey anti-Mouse IgG (H + L) Secondary Antibody, Alexa Fluor 647	Thermo Fisher Scientific	Cat #A-31571; RRID: AB_162542
DAPI	Santa Cruz	Cat# D1306; RRID: AB_2629482

**Bacterial and virus strains**		

ZIKV^U^ (West Africa strain, MR766)	ZeptoMetrix	# 0810521CF
ZIKV^PR^ (Puerto Rico strain, PRVABC59)	ZeptoMetrix	# 0810521CF

**Chemicals, peptides, and recombinant proteins**		

Y-27632	MedchemExpress	#HY-10583
Recombinant Human bFGF Protein	Peprotech	#100-18B-500UG
Recombinant Human BMP-4 Protein	R & D Systems	#314-BP
StemFlex	Gibco Thermo Fisher	#A3349401
Knockout Serum Replacement	Gibco Thermo Fisher	#10828028
DMEM (high glucose)	Gibco Thermo Fisher	#11-965-118
Penicillin-Streptomycin (5,000 U/mL)	Gibco Thermo Fisher	#15070063
GlutaMAX Supplement	Thermo Fisher Scientific	#35050079
MEM Non-Essential Amino Acids Solution (100X)	Gibco Thermo Fisher	#11140050
Accutase	Stem Cell Technologies	# 07920
ReleSR	Stem Cell Technologies	# 05872
Matrigel	Corning	#354234

**Deposited data**		

Original western blot images	This paper (Elsevier’s Mendeley Data repository)	https://doi.org/10.17632/9y6cbwtwbg.1

**Experimental models: Cell lines**		

293T	ATCC	#CRL-11268
Vero E6	ATCC	#CRL-1586
irradiated CF1 Mouse Embryonic Fibroblasts	Thermo Fisher Scientific	#A34180

**Experimental models: Organisms/strains**		

SCID-beige mice	The Jackson Laboratory	NA

**Recombinant DNA**		

pLV[Exp]-EGFP:T2A:Puro-EF1A > hNDUFA4	VectorBuilder	VB900004-0599xvh
pLV[shRNA]-EGFP:T2A:Puro-U6>hNDUFA4[shRNA#1]	VectorBuilder	VB900052-8762rzm
pLV[shRNA]-EGFP:T2A:Puro-U6>hNDUFA4[shRNA#2]	VectorBuilder	VB900052-8767wwk

**Software and algorithms**		

Adobe illustrator CC2017	Adobe	https://www.adobe.com/product/photoshop.html
Graphpad Prism 8	Graphpad software	https://www.graphpad.com

### Resource availability

#### Lead contact

Further information and requests for resources and reagents should be directed to and will be fulfilled by the Lead Contact, Shuibing Chen (shc2034@med.cornell.edu).

#### Materials availability

*NDUFA4*^*−/−*^, WT iPSCs, iPSCs carrying risk (*G/G*; *C/C*) or non-risk (*T/T*; *T/T*) alleles, and *NDUFA4*^Δ^, WT_Δ iPSCs are available upon request under an appropriate Material Transfer Agreement.

### Experimental model and subject details

#### The age and species/strains of mice

Six to eight weeks of age matched mice were used for mouse studies. Males were used for animal studies.

#### *In vivo* animal studies

All animal work was conducted in agreement with NIH guidelines and approved by the Weill Cornell Medicine Institutional Animal Care and Use Committee and the Institutional Biosafety Committee.

#### iPSC cell lines

All hiPSC experiments have been approved by Weill Cornell Medicine Human Embryonic Stem Cell Research Oversight Committee. The gender of iPSCs are: iPSC#1: Female; iPSC#41: Male; iPSC#44: Female; iPSC#15: Male; iPSC#17: Female; iPSC#19: Female. Cells were tested negative for mycoplasma contamination.

### Method details

#### hiPSC culture and infection

hiPSCs were grown on matrigel-coated plates using StemFlex medium (Thermo Fisher Scientific, #A3349401). Cells were maintained at 37°C with 5% CO_2_. ZIKV^PR^ (PRVABC59) strain and ZIKV^U^ (MR766) strain were obtained from ZeptoMetrix and titered by plaque assay using Vero cells. iPSCs were infected with ZIKV (ZIKV^U^, MOI =0.15; ZIKV^PR^ strain MOI = 1) for 2 h and changed to virus-free medium. At 72 hpi, cells were fixed, stained with the antibody against the ZIKV envelope protein (ZIKV-E), and analyzed by qRT-PCR.

#### Construction of NDUFA4 knockdown cell lines

The control shRNA and NDUFA4 specific shRNAs were obtained from VectorBuilder (VB900052-8767wwk and VB900052-8775ugm). The shRNA plasmids were transfected into 293T cells together with pMD2G and psPAX2 plasmids using calcium phosphate transfection. Lentivirus was collected and concentrated using Lenti-X Concentrator (Clontech) at 48 and 72 hpi. Then, iPSC line #1 and iPSC line #41 were infected with lentivirus for 48 h and sorted based on GFP expression.

#### Animal transplantation and infection

3.0 × 10^6^ WT or *NDUFA4*^−/−^ iPSCs were subcutaneously injected into 6-8-week-old male SCID-beige mice. Two weeks later, the mice were inoculated with ZIKV virus (5 × 10^5^ PFU) by intraperitoneal injection. Five days after virus infection, the mice were euthanized. Xenografts were isolated, fixed in 4% PFA for two days and then transferred to 30% sucrose, followed by snap-freezing in O.C.T (Fisher Scientific).

#### Generation and infection of trophectoderm cells

Human iPSCs were differentiated into trophectoderm cells using our previously reported protocol.^9^ Briefly, hiPSCs were cultured to reach 80% confluent and dissociated to single cells with Accutase (Innovative Cell Technologies, #AT-104). Then, cells were seeded at 2.0 × 10^5^ cells/well on 6-well plates coated with matrigel (100X, Corning, #354277) and cultured in StemFlex medium (Thermo Fisher Scientific, #A3349401) supplemented with 10 μM ROCK inhibitor Y-27632 (R&D Systems, #1254). The day after plating, trophectoderm cell differentiation was initiated by changing the culture medium to MEF conditional medium supplemented with 4 ng/mL bFGF (Peprotech, 100-18B). Then, cells were cultured in MEF conditional medium supplemented with 100 ng/mL BMP4 for another 4 days. On day 4, trophectoderm cells were harvested enzymatically with Accutase (Innovative Cell Technologies, #AT-104) and replated onto matrigel-coated 96-well plates or 24-well plates. iPSC-derived trophectoderm cells were infected with ZIKV (ZIKV^U^, MOI =0.15; ZIKV^PR^ strain MOI = 1) for 2 h and changed to virus-free medium. At 72 hpi, cells were collected for analysis.

MEF conditional medium was prepared using irradiated CF1 Mouse Embryonic Fibroblasts (Thermo Fisher Scientific, #A34180). MEF conditional medium contains DMEM high glucose (Gibco Thermo Fisher, #11-965-118) supplemented with 10% Knockout Serum Replacement (Thermo Fisher, #10828028), 1×NEAA (Thermo Fisher, #11140050), 1×Glutamax (Thermo Fisher, #35050079) and 1×PenStrep (Penicillin, #15140163).

#### qRT-PCR

Total RNA samples were prepared from cells using TRIzol reagent (Qiagen, #74136) and reverse transcribed (RT) with high-capacity cDNA reverse transcription Kit supplemented with RNase inhibitor (Thermo Fisher, # 4374966). For the quantification of (+) and (−) strands of ZIKV vRNAs, strand specific primers were used in RT. Human β-Actin was employed as an internal reference. PrimeTime Gene Expression 2X Master Mix (IDT, #1055772) and probes for ZIKV vRNA and human β-Actin were used to perform qPCR reactions. For quantification of genes, random primers were used in RT. Human β-Actin was employed as an internal reference. The sequences of primers/probes are provided in Table S1.

#### Virus titer assay

Supernatants were collected from ZIKV infected cell cultures and then diluted serially from 10- to 10^8^-fold. Vero cells were maintained in DMEM medium plus 10% fetal bovine serum. Cells were plated into 96-well plates at a density of 25,000 cells/well and then were infected with diluted supernatants in 96-well plates for 2 h. After infection, medium was replaced with semi-solid medium of alpha-MEM containing 10% fetal bovine serum and 1% methylcellulose. Two days after infection, cells were fixed with 4% PFA and stained with anti-ZIKV-E antibody.

#### Immunohistochemistry

Cells were fixed in 4% PFA for 20 min at room temperature, blocked in Mg^2+^/Ca^2+^ free PBS plus 5% horse serum and 0.2% Triton-X for 1 hour at room temperature, and then incubated with primary antibody at 4°C overnight. The information for primary antibodies is provided in Table S2. Secondary antibodies included donkey anti-mouse, goat, rabbit or chicken antibodies conjugated with Alexa-Fluor-488, Alexa-Fluor-594 or Alexa-Fluor-647 fluorophores (1:500, Life Technologies). Nuclei were counterstained by DAPI. Images were acquired using an LSM 880 Laser Scanning Confocal Microscope and processed with Zen software. Quantification was performed using ImageJ (NIH) software.

#### Western blotting

Cells were collected in Pierce RIPA buffer (Thermo Fisher Scientific) plus HALT protease inhibitor cocktail (1:100) (Thermo Fisher Scientific) and lysates loaded on 12% NuPage Bis-Tris pre-cast gels (Thermo Fisher Scientific). After separation by electrophoresis, proteins were transferred to 0.2 μm nitrocellulose membranes (Thermo Fisher Scientific). Membranes were blocked with 5% milk in TBS +0.1% Tween and incubated with primary antibody overnight. Information for primary antibodies is provided in Table S2. Membranes were washed and incubated with secondary antibody for 1 h at room temperature in 5% milk-TBS-0.1% Tween and developed using SuperSignal West Pico PLUS chemiluminescent substrate (Thermo Fisher Scientific). Human β-Actin was employed as an internal reference.

#### Flow cytometry analysis

hiPSC-derived trophectoderm cells were dissociated with Acctuase into single cells and washed with PBS twice, then cells were fixed and permeabilized using Fixation/Permeabilization Solution Kit (BD Biosciences). Cells were incubated with primary antibody at 4°C overnight and secondary antibodies at RT for 1 h, followed by washing twice and flow cytometry analysis. The information for primary antibodies and secondary antibodies were provided in Table S2.

### Quantification and statistical analysis

Data are shown as mean ± SD. For comparisons with a common control, we used the one-way ANOVA followed by a *Dunnett’s* post hoc test for multiple testing correction. For low permissive and permissive cell line comparisons, we averaged the 3 technical replicates within each cell line, then used the averages for an unpaired two-tailed Student’s t-test. For the comparisons of two phenotypes and two colonies, we used two-way ANOVA analysis followed by *Tukey’s* post hoc test for multiple testing correction. N=3 independent biological replicates were used for all experiments unless otherwise indicated. n.s. indicates a non-significant difference. ∗p<0.05, ∗∗p<0.01 and ∗∗∗p<0.001. For statistical analyses, we used GraphPad Prism 8.4.1 software.

## Data Availability

• Original western blot images have been deposited at Mendeley and are publicly available as of the date of publication. The DOI is listed in the key resources table. Microscopy data reported in this paper will be shared by the lead contact upon request. • There is no newly generated code to report. • Any additional information required to reanalyze the data reported in this paper is available from the lead contact upon request.
