# Supplementary material for: Isogenic human trophectoderm cells demonstrate the role of NDUFA4 and associated variants in ZIKV infection
Source: iScience. 2023 May 29;26(7):107001. doi: 10.1016/j.isci.2023.107001 (PMC10391681; doi:10.1016/j.isci.2023.107001)
Supplement: Document S1. Figures S1–S6 and Tables S1 and S2 [file mmc1.pdf]

iScience, Volume *26*

Supplemental information

Isogenic human trophectoderm cells demonstrate the role of *NDUFA4* and associated variants in ZIKV infection

Liuliu Yang, Yuling Han, Ting Zhou, Lauretta A. Lacko, Mohsan Saeed, Christina Tan, Ron Danziger, Jiajun Zhu, Zeping Zhao, Clare Cahir, Alice Maria Giani, Yang Li, Xue Dong, Dorota Moroziewicz, The NYSCF Global Stem Cell Array® Team, Daniel Paull, Zhengming Chen, Aaron Zhong, Scott A. Noggle, Charles M. Rice, Qibin Qi, Todd Evans, and Shuibing Chen

Figure S1

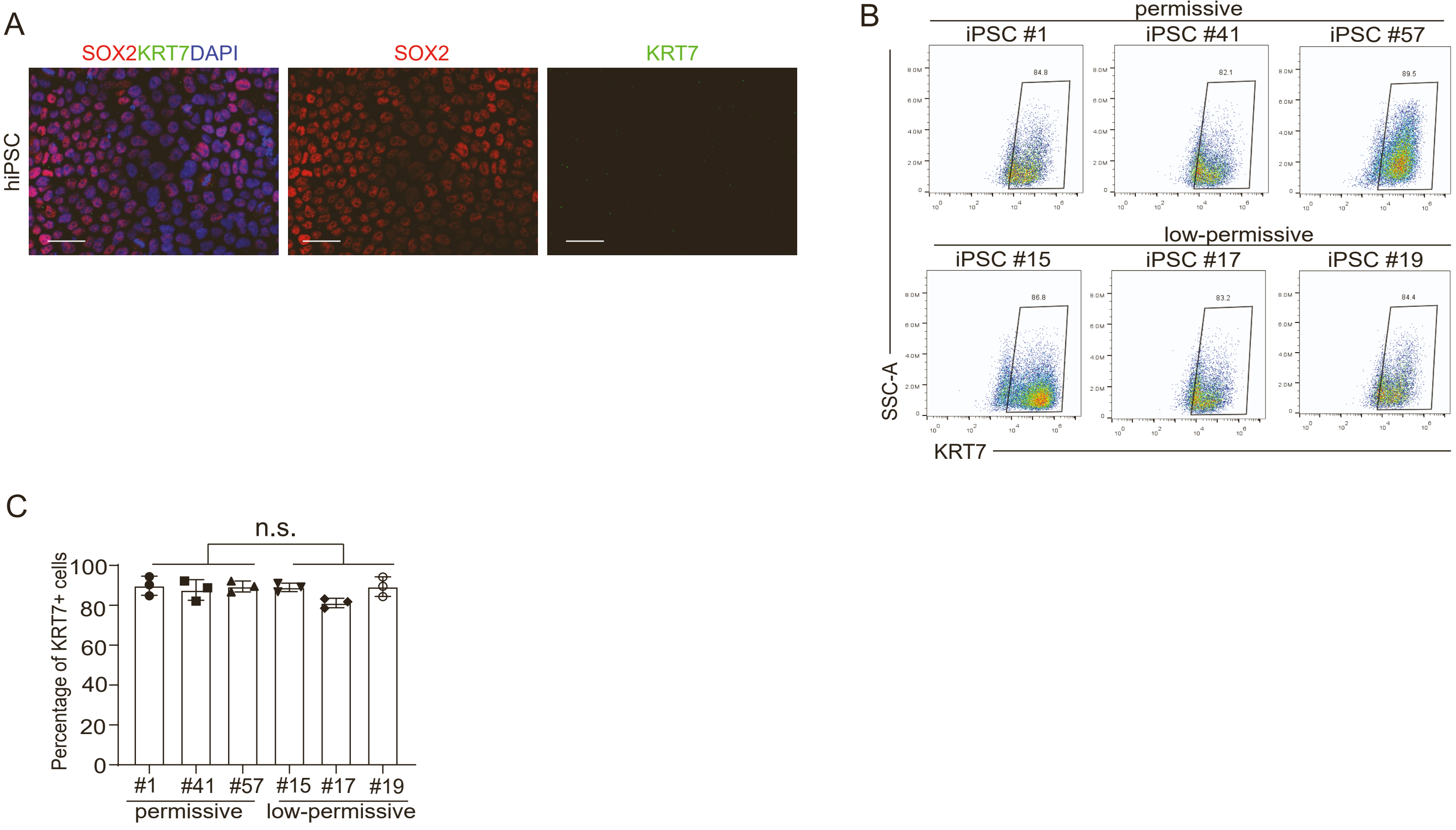
A
SOX2KRT7DAPI
SOX2
KRT7
hiPSC
B
permissive
iPSC #1
iPSC #41
iPSC #57
84.8
82.1
89.5
low-permissive
iPSC #15
iPSC #17
iPSC #19
86.8
83.2
84.4
SSC-A
KRT7
C
n.s.
Percentage of KRT7+ cells
100
80
60
40
20
0
#1
#41
#57
#15
#17
#19
permissive
low-permissive

SUPPLEMENTAL FIGURES

Figure S1, related to Figure 1. Different background hiPSCs are equally efficient at generating trophectoderm cells.

(A) Representative confocal images of SOX2 and KRT7 staining in hiPSCs. Scale bar=50 μm.

(B and **C)** Flow cytometry analysis (B) and quantification (C) of KRT7 staining in trophectoderm cells derived from permissive cell lines: iPSC #1, iPSC #41 and iPSC #57 or low permissive cell lines: iPSC #15, iPSC #17 and iPSC #19.

Data are representative of at least three independent experiments. For *P* values, we averaged the 3 technical replicates within each cell line, then used the averages for an unpaired two-tailed *Student's t*-test. n.s. no significance.

Figure S2

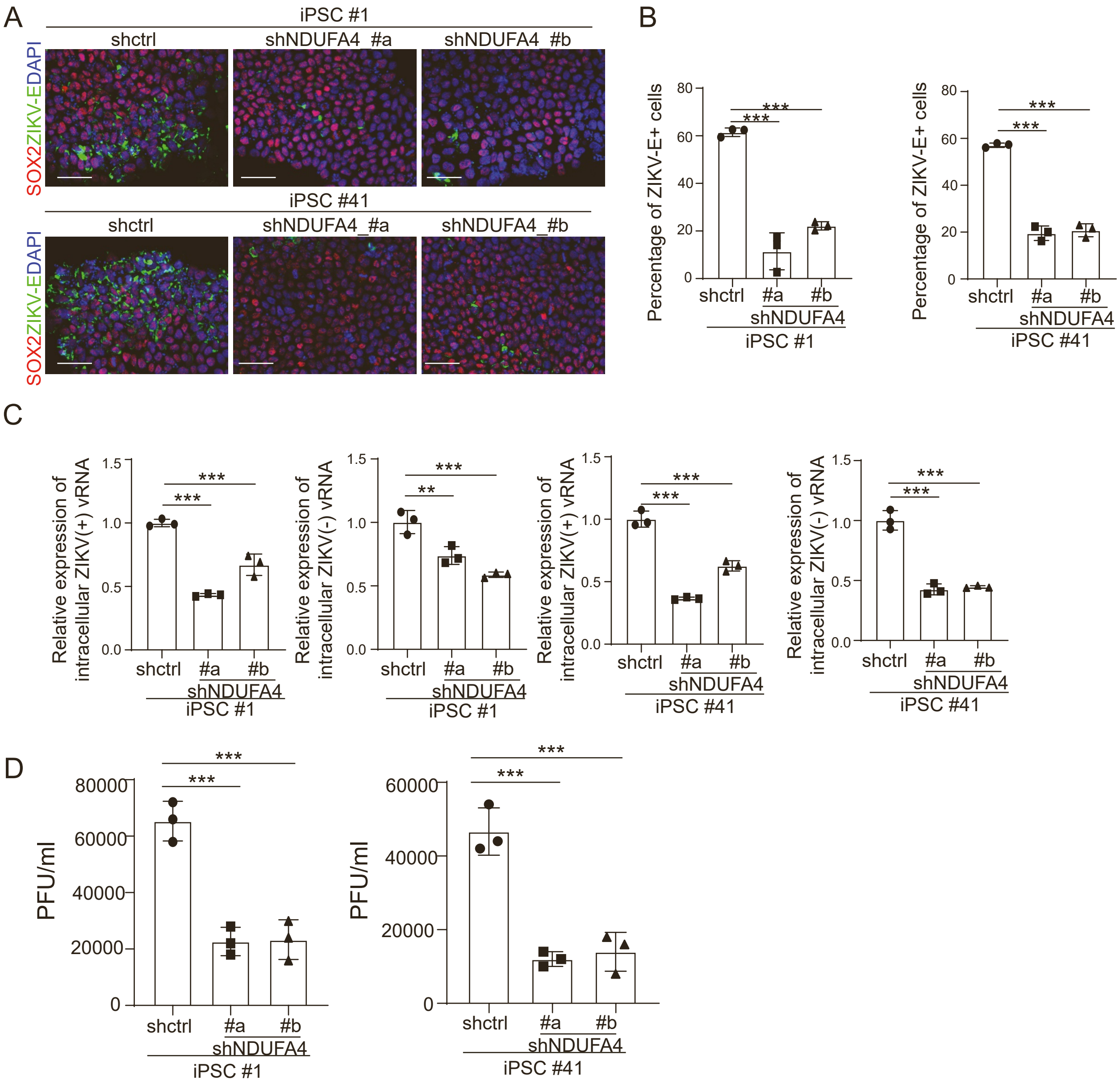
A
iPSC #1
shctrl
shNDUFA4_#a
shNDUFA4_#b
SOX2ZIKV-EDAPI
iPSC #41
B
Percentage of ZIKV-E+ cells
shNDUFA4
C
Relative expression of intracellular ZIKV(+) vRNA
Relative expression of intracellular ZIKV(-) vRNA
D
PFU/ml
#a
#b
***
**

Figure S2, related to Figure 2. Knockdown of *NUDFA4* decreases ZIKV permissiveness to infection.

(A and **B)** Representative confocal images (A) and the quantification (B) of ZIKV-E and SOX2 staining in NDUFA4 knockdown lines of iPSC #1 (shctrl, shNDUFA4 #a, shNDUFA4 #b) and iPSC #41 (shctrl, shNDUFA4 #a, shNDUFA4 #b) at 72 hpi ($ZIKV^{U}$, MOI=0.15). Scale bar=50 µm.

(C) qRT-PCR analysis of (+) or (-) ZIKV vRNA strands in NDUFA4 knockdown lines of iPSC #1 (shctrl, shNDUFA4 #a, shNDUFA4 #b) and iPSC #41 (shctrl, shNDUFA4 #a, shNDUFA4 #b) at 72 hpi ($ZIKV^{U}$, MOI=0.15).

(D) Viral titers of ZIKV virus in the supernatant of NDUFA4 knockdown cell lines of iPSC #1 (shctrl, shNDUFA4 #a, shNDUFA4 #b) or iPSC #41 (shctrl, shNDUFA4 #a, shNDUFA4 #b) at 72 hpi ($ZIKV^{U}$, MOI=0.15) quantified by plaque assay.

Data are representative of at least three independent experiments. *P* values were calculated by one-way ANOVA followed by a *Dunnet*t's post hoc test with a common control for multiple testing correction. **$P < 0.01$, ***$P < 0.001$.

Figure S3

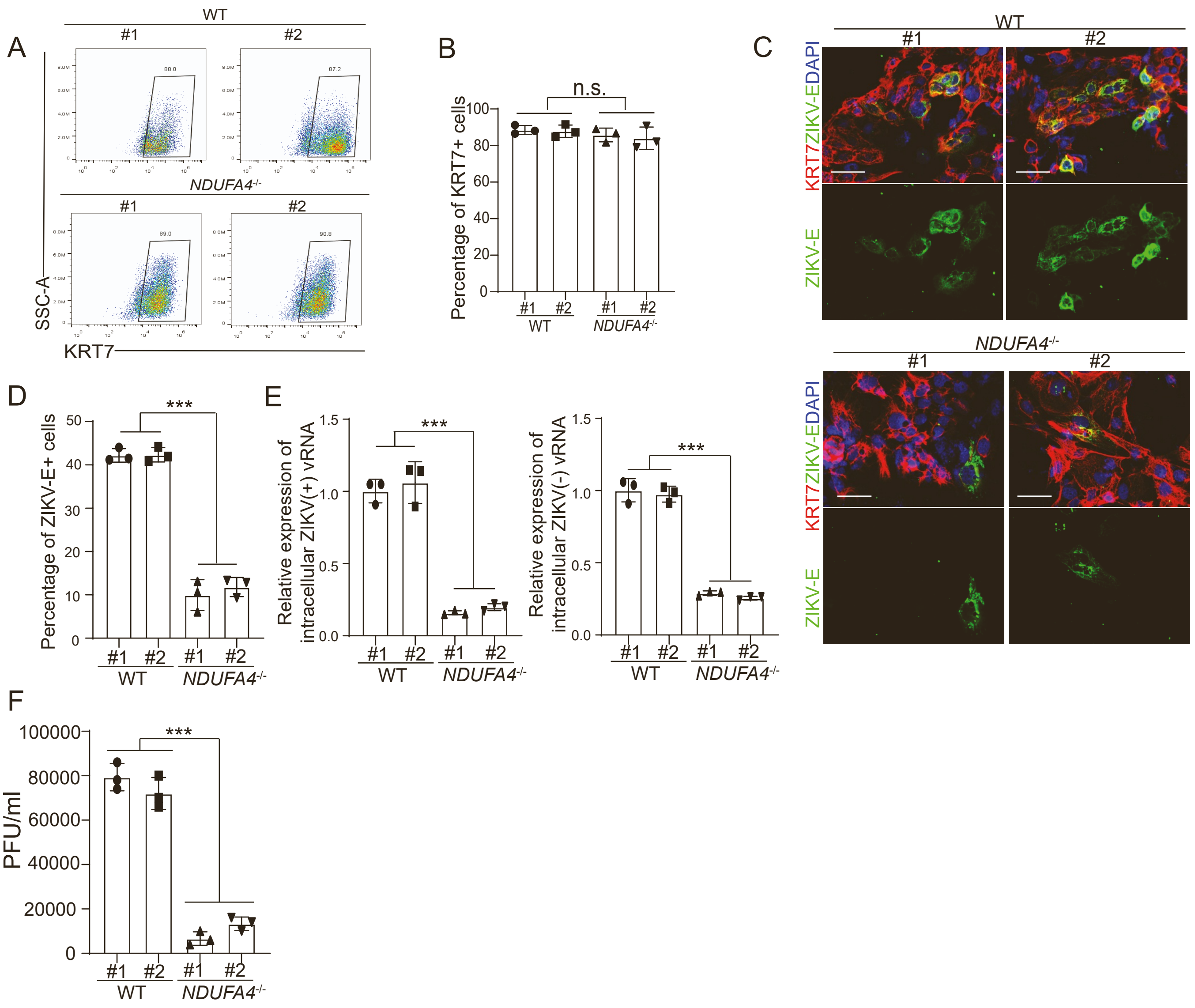
A
WT
#1
#2
NDUFA4-/-
#1
#2
SSC-A
KRT7
B
n.s.
Percentage of KRT7+ cells
#1 #2
WT
#1 #2
NDUFA4-/-
C
WT
#1
#2
KRT7ZIKV-EDAPI
ZIKV-E
NDUFA4-/-
#1
#2
KRT7ZIKV-EDAPI
ZIKV-E
D
***
Percentage of ZIKV-E+ cells
#1 #2
WT
#1 #2
NDUFA4-/-
E
***
Relative expression of intracellular ZIKV(+) vRNA
#1 #2
WT
#1 #2
NDUFA4-/-
***
Relative expression of intracellular ZIKV(-) vRNA
#1 #2
WT
#1 #2
NDUFA4-/-
F
***
PFU/ml
#1 #2
WT
#1 #2
NDUFA4-/-

Figure S3, related to Figure 3. The expression of NDUFA4 is associated with ZIKV infection in trophectoderm cells.

(A and **B)** Flow cytometry analysis (A) and the quantification (B) of $KRT7^+$ cells in trophectoderm cells derived from WT or *NDUFA4*$^{-/-}$ hiPSCs.

(C and **D)** Representative images (C) and the quantification (D) of ZIKV-E staining in $KRT7^+$ trophectoderm cells derived from WT or *NDUFA4*$^{-/-}$ hiPSCs at 72 hpi ($ZIKV^U$, MOI=0.15). Scale bar=50 μm.

(E) qRT-PCR analysis of (+) and (-) ZIKV vRNA strands in trophectoderm cells derived from WT or *NDUFA4*$^{-/-}$ hiPSCs at 72 hpi ($ZIKV^U$, MOI=0.15).

(F) Viral titers of ZIKV virus in the supernatant of trophectoderm cells derived from WT or *NDUFA4*$^{-/-}$ hiPSCs at 72 hpi ($ZIKV^U$, MOI=0.15) quantified by plaque assay.

Data are representative of at least three independent experiments. *P* values were calculated by two-way ANOVA analysis; n.s. no significance, ***$P < 0.001$.

Figure S4

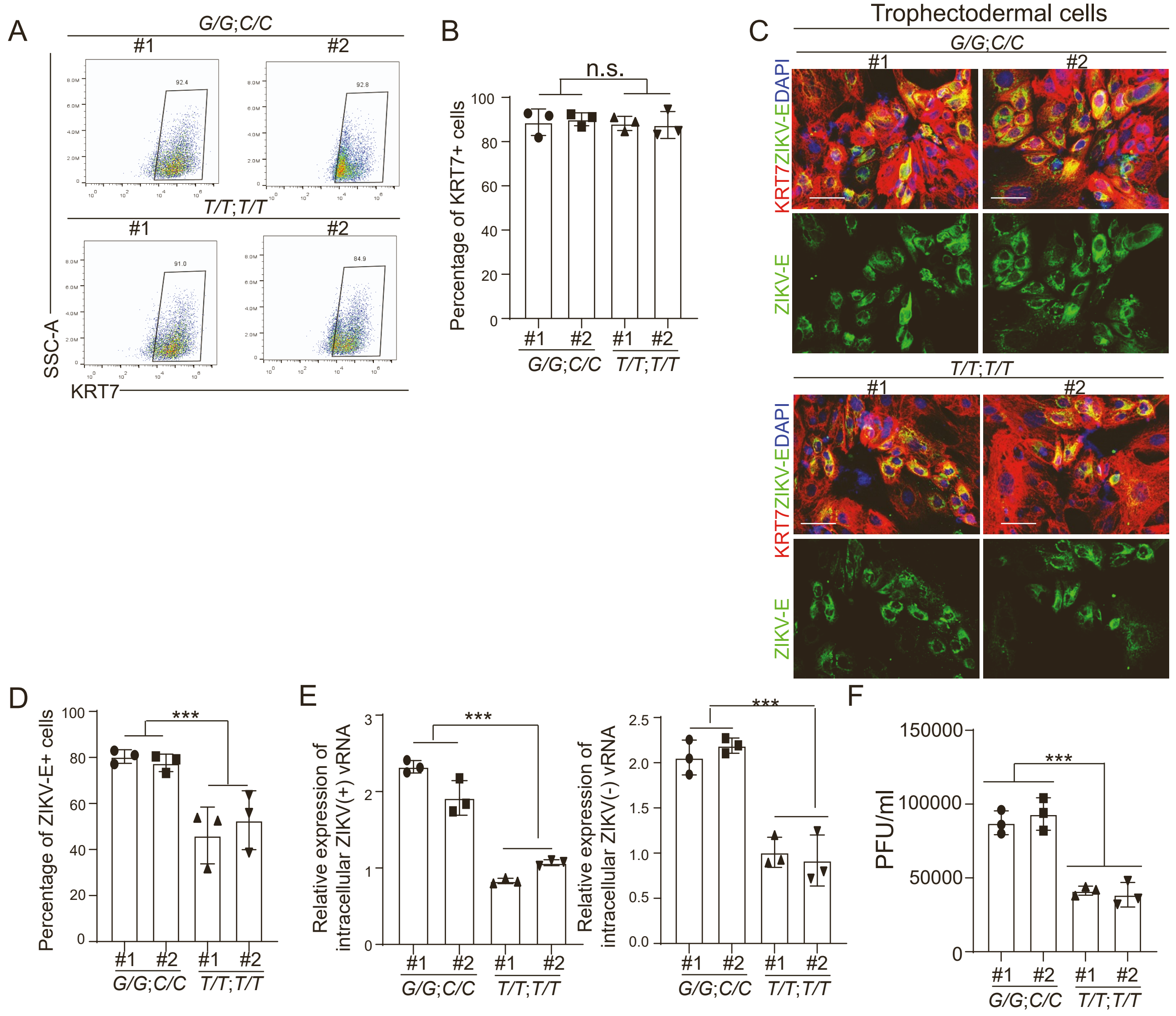

A
G/G;C/C
#1
#2
T/T;T/T
SSC-A
KRT7
B
n.s.
Percentage of KRT7+ cells
G/G;C/C
T/T;T/T
C
Trophectodermal cells
KRT7ZIKV-EDAPI
ZIKV-E
D
Percentage of ZIKV-E+ cells
***
E
Relative expression of intracellular ZIKV(+) vRNA
Relative expression of intracellular ZIKV(-) vRNA
F
PFU/ml

Figure S4, related to Figure 4. SNPs for risk alleles of *NDUFA4* promote the infection of ZIKV virus in trophectoderm cells.

(A and **B)** Flow cytometry analysis (A) and the quantification (B) of $KRT7^{+}$ cells in trophectoderm cells differentiated from hiPSC lines carrying risk (*G/G*; *C/C*) or non-risk (*T/T*; *T/T*) alleles.

(C and **D)** Representative images (C) and the quantification (D) of ZIKV-E staining in $KRT7^{+}$ trophectoderm cells derived from hiPSC lines carrying risk (*G/G*; *C/C*) or non-risk (*T/T*; *T/T*) alleles at 72 hpi ($ZIKV^{U}$, MOI=0.15). Scale bar=50 μm.

(E) qRT-PCR analysis of (+) and (-) ZIKV vRNA strands in trophectoderm cells derived from hiPSC lines carrying risk (*G/G*; *C/C*) or non-risk (*T/T*; *T/T*) alleles at 72 hpi ($ZIKV^{U}$, MOI=0.15).

(F) Viral titers of ZIKV virus in the supernatant of trophectoderm cells derived from hiPSCs carrying risk (*G/G*; *C/C*) or non-risk (*T/T*; *T/T*) alleles at 72 hpi ($ZIKV^{U}$, MOI=0.15) quantified by plaque assay.

Data are representative of at least three independent experiments. *P* values were calculated by two-way ANOVA analysis; n.s. no significance, ***$P < 0.001$.

Figure S5

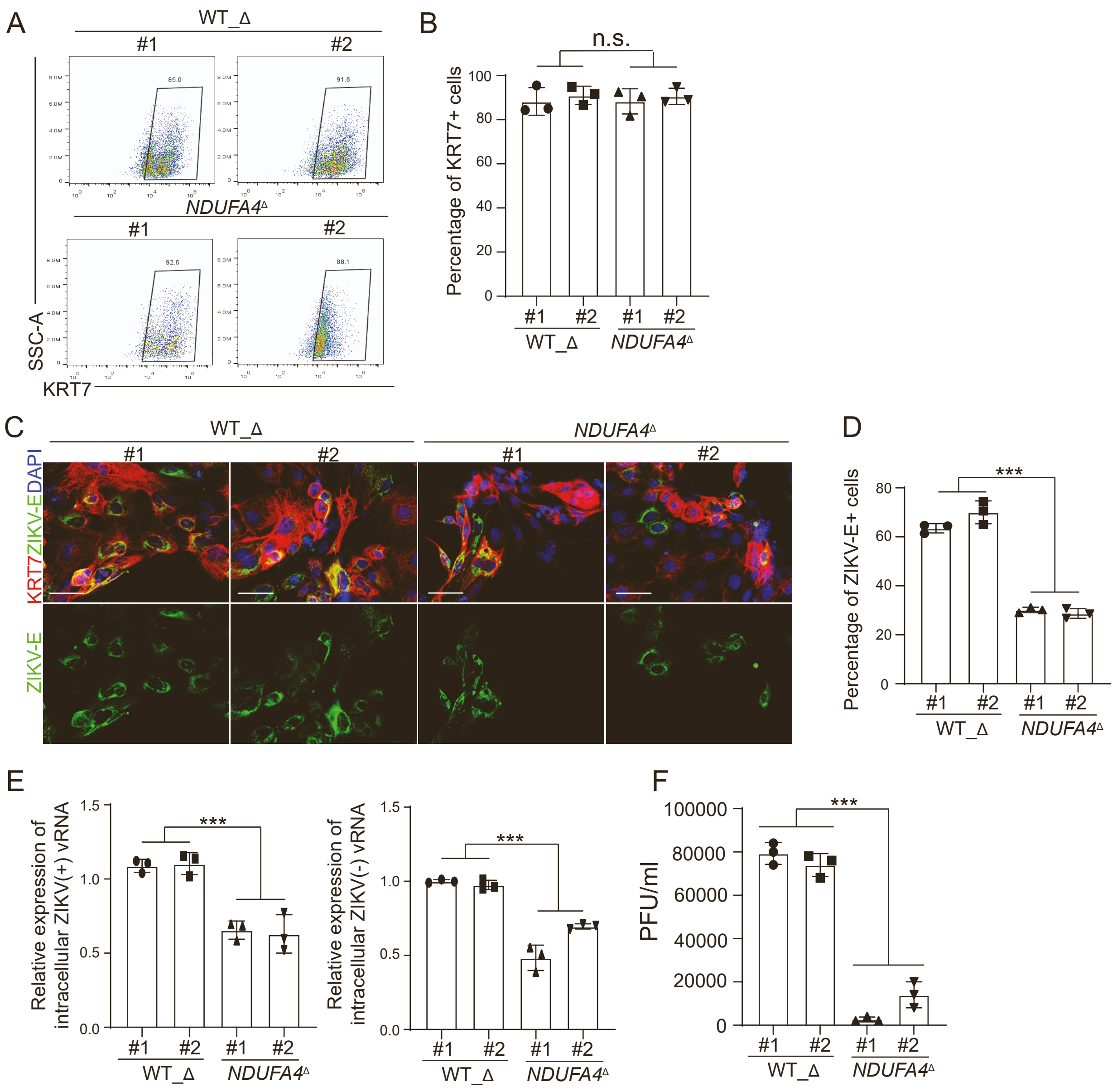
A
WT_Δ
#1
#2
85.0
91.6
NDUFA4Δ
92.6
88.1
SSC-A
KRT7
B
n.s.
Percentage of KRT7+ cells
100
80
60
40
20
0
#1
#2
WT_Δ
NDUFA4Δ
C
KRT7ZIKV-EDAPI
ZIKV-E
D
***
Percentage of ZIKV-E+ cells
E
Relative expression of intracellular ZIKV(+) vRNA
Relative expression of intracellular ZIKV(-) vRNA
1.5
1.0
0.5
0.0
F
PFU/ml
100000
80000
60000
40000
20000

Figure S5, related to Figure 5. Deletion of the *cis*-regulatory region decreases the sensitivity of ZIKV infection in trophectoderm cells.

(A and **B)** Flow cytometry analysis (A) and the quantification (B) of $KRT7^{+}$ cells in trophectoderm cells derived from WT_Δ or $NDUFA4^{\Delta}$ hiPSCs.

(C and **D)** Representative images (C) and the quantification (D) of ZIKV-E staining in $KRT7^{+}$ trophectoderm cells derived from WT_Δ or $NDUFA4^{\Delta}$ hiPSCs at 72 hpi ($ZIKV^{U}$, MOI=0.15). Scale bar=50 μm.

(E) qRT-PCR analysis of (+) or (-) ZIKV vRNA strands of trophectoderm cells derived from WT_Δ or $NDUFA4^{\Delta}$ hiPSCs at 72 hpi ($ZIKV^{U}$, MOI=0.15).

(F) Viral titers of ZIKV virus in the supernatant of trophectoderm cells derived from WT_Δ or $NDUFA4^{\Delta}$ hiPSCs at 72 hpi ($ZIKV^{U}$, MOI=0.15) quantified by plaque assay.

Data are representative of at least three independent experiments. *P* values were calculated by two-way ANOVA analysis; n.s. no significance and ***$P < 0.001$.

Figure S6

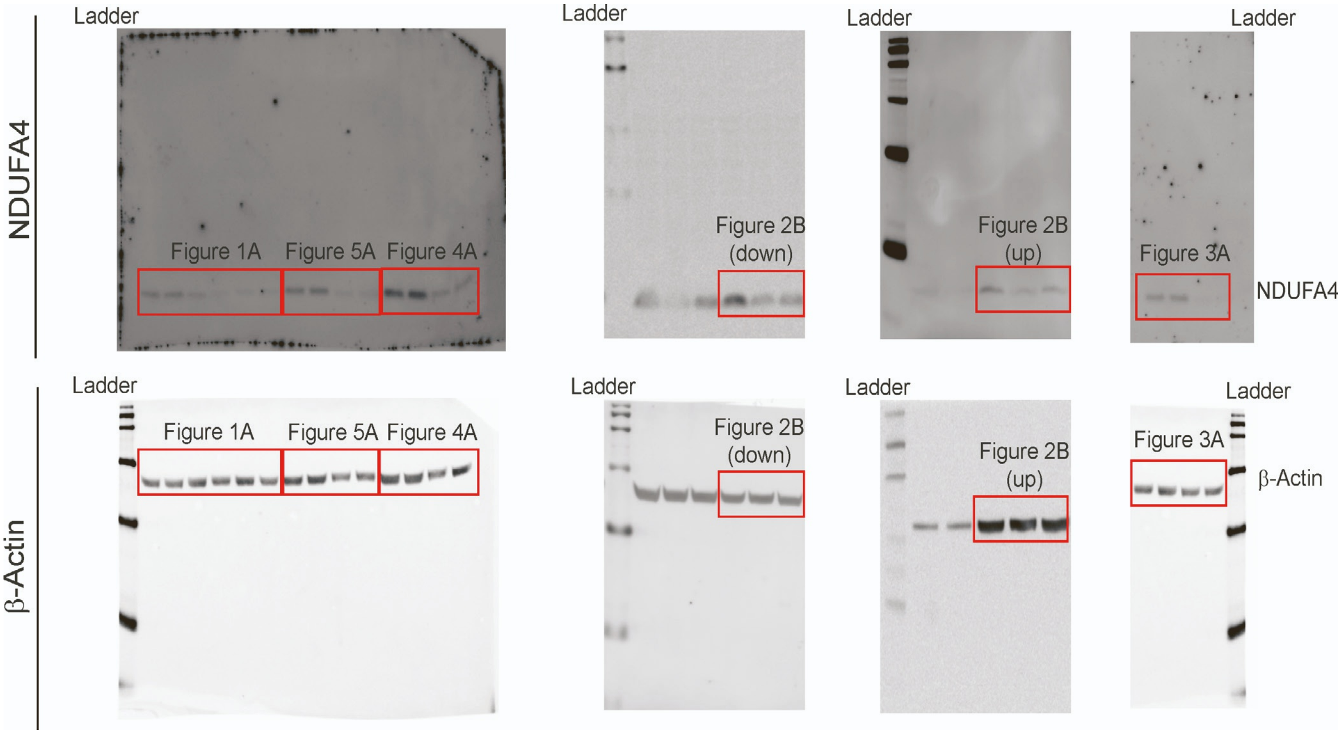

NDUFA4
Ladder
Figure 1A
Figure 5A
Figure 4A
Ladder
Figure 2B
(down)
Ladder
Figure 2B
(up)
Figure 3A
Ladder
NDUFA4
β-Actin
Ladder
Figure 1A
Figure 5A
Figure 4A
Ladder
Figure 2B
(down)
Ladder
Figure 2B
(up)
Figure 3A
Ladder
β-Actin

Figure S6, related to figure1-5. Raw data of western blotting.

Table S1. Primers/probes used for Sanger sequencing, PCR and qRT-PCR, Related to STAR Methods.

**Primer Name**	**Sequence**
ZIKV (+) vRNA-RT	*TACTTGTACAGCTCGTCCATGCCACTAACGTTCT TTTGCAGACAT*
ZIKV (+) vRNA-PCR forward	*CCGCTGCCCAACACAAG*
ZIKV (+) vRNA-PCR reverse	*TACTTGTACAGCTCGTCCATG*
ZIKV (+) vRNA-PCR probe	*5'-/56-FAM/AGCCTACCT/ZEN/TGACAAGCAATCAGACA CTCAA/3IABkFQ/-3'*
ZIKV (-) vRNA-RT	*AACAGCCACAACGTCTATATCCCGCTGCCCAAC ACAAG*
ZIKV (-) vRNA-PCR forward	*AACAGCCACAACGTCTATATC*
ZIKV (-) vRNA-PCR reverse	*CCACTAACGTTCTTTTGCAGACAT*
ZIKV (-) vRNA-PCR probe	*5'-/56-FAM/TTGAGTGTC/ZEN/TGATTGCTTGTCAAGGTA GGCT/3IABkFQ/-3'*
Human *ACTB*-RT	*CCTGGATAGCAACGTACATGG*
Human *ACTB*-PCR forward	*CCTTGCACATGCCGGAG*
Human *ACTB*-PCR reverse	*ACAGAGCCTCGCCTTTG*
Human *ACTB*-PCR probe	*5'-/5HEX/TCATCCATG/ZEN/GTGAGCTGGCGG/3IABk FQ/-3'*
Human *ACTB*-PCR forward	*ACCTTCTACAATGAGCTGCG*
Human *ACTB*-PCR reverse	*CCTGGATAGCAACGTACATGG*
Human *NDUFA4*-PCR forward	*CATCGGTCAGGCCAAGAA*
Human *NDUFA4*-PCR reverse	*GGGCTCTGGGTTATTTCTGTC*
Human *ISG15* forward	*CGCAGATCACCCAGAAGATCG*
Human *ISG15* reverse	*TTCGTCGCATTTGTCCACCA*
Human *IRF7* forward	*CCCACGCTATACCATCTACCT*
Human *IRF7* reverse	*GATGTCGTCATAGAGGCTGTTG*

Table S2. Antibodies used for immunocytochemistry, intracellular flow cytometry analysis and western blotting analysis, Related to STAR Methods.

Usage	Antibody	Clone #	Host	Catalog #	Vendor	Dilution
Immunocytochemistry	Anti-Flavivirus group antigen	D1-4G2-4-15	Mouse	GTX57154	GeneTex	1:100
Immunocytochemistry	Anti-Flavivirus group antigen	D1-4G2-4-15	Mouse	MAB10216-I-100UG	Millipore	1:1000
Western blot	Anti-NDUFA4	Polyclonal	Rabbit	ab129752	Abcam	1:200
Immunocytochemistry	Anti-SOX2	D6D9	Rabbit	3579	Cell Signaling	1:400
Immunocytochemistry	Keratin7 Rabbit mAb	D1E4	Rabbit	4465	Cell Signaling	1:200
Immunocytochemistry	Alexa Fluor 594 anti-Mouse IgG (H+L) Secondary Antibody	Polyclonal	Donkey	A-21203	Thermo Scientific	1:500
Immunocytochemistry	Alexa Fluor 488 anti-Rabbit IgG (H+L) Secondary Antibody	Polyclonal	Donkey	A-21206	Thermo Scientific	1:500
Western blot	Anti-β actin	15G5A11/E2	Mouse	MA1-140	Thermo Scientific	1:5000
Western blot	680RD Donkey anti-Mouse IgG Secondary Antibody	Polyclonal	Donkey	926-68072	Li-Cor	1:15000
Western blot	800CW Donkey anti-Rabbit IgG	Polyclonal	Donkey	926-32213	Li-Cor	1:15000

	Secondary Antibody					
